# Cellular Responses to Tartrazine and Sulfanilic Acid Exposure in BEAS‐2B Cells: Viability, Apoptosis, and DNA Damage

**DOI:** 10.1002/jat.70174

**Published:** 2026-03-18

**Authors:** Merve Baysal, Abdullah Burak Karaduman, Büşra Korkut Çelikateş, Merve Güven, Özlem Atlı‐Eklioğlu, Sinem Ilgın

**Affiliations:** ^1^ Department of Pharmaceutical Toxicology, Faculty of Pharmacy Anadolu University Eskişehir Turkey

**Keywords:** apoptosis, BEAS‐2B, cytotoxicity, DNA damage, sulfanilic acid, tartrazine

## Abstract

Tartrazine is a synthetic azo dye widely used in food, pharmaceutical, and cosmetic products, resulting in extensive human exposure, while its toxicity and that of its primary metabolite, sulfanilic acid, remain controversial. Considering the reported association of tartrazine with hypersensitivity and allergic‐like reactions, human bronchial epithelial BEAS‐2B cells, which are relevant for airway and allergy‐related responses, were selected as the in vitro model. This study investigated the effects of tartrazine and sulfanilic acid on cell viability, apoptosis‐related responses, and DNA damage. Cell viability was evaluated using the MTT and neutral red uptake assays. Apoptotic responses were assessed by annexin V/propidium iodide staining, mitochondrial membrane potential analysis, and caspase‐3/7 activity measurement. DNA damage was examined using the alkaline comet assay. Tartrazine reduced cell viability in a concentration‐ and time‐dependent manner in the MTT assay, whereas sulfanilic acid showed minimal cytotoxic effects. Both compounds increased apoptotic cell populations at selected concentrations. Tartrazine induced mitochondrial membrane potential depolarization, while caspase‐3/7 activity decreased at higher concentrations of both compounds. No significant DNA strand breaks were detected under the experimental conditions applied. These findings suggest that tartrazine and sulfanilic acid predominantly affect cell viability and apoptosis‐related processes in airway epithelial cells without detectable DNA strand breaks at the tested concentrations.

## Abstract 2

Tartrazine is a widely used synthetic azo dye, and its toxicity, together with that of its primary metabolite sulfanilic acid, remains debated. Given the reported allergy‐related effects of tartrazine, human bronchial epithelial BEAS‐2B cells were selected as an in vitro model. The effects of both compounds on cell viability, apoptosis‐related endpoints, and DNA damage were evaluated. Tartrazine induced concentration‐ and time‐dependent cytotoxicity, whereas sulfanilic acid showed limited effects. Both compounds increased apoptotic cell populations without detectable DNA strand breaks.

## 1 Introduction

Artificial colorants are widely incorporated into foods to enhance visual attractiveness or to mask natural and technological variations in appearance. Among these additives, azo dyes constitute the most extensively used class of synthetic colorants in the global food industry (Ramos‐Souza et al. 2023). Azo dyes have a wide application spectrum, spanning the food, pharmaceutical, cosmetic, paper, textile, and leather industries. Notably, approximately 65% of these compounds are employed as food additives in products such as soft drinks, jams, candies, and pickled foods (Barciela et al. 2023).

Tartrazine (E102) is a bright yellow synthetic azo dye with a broad range of applications comparable with other azo colorants and is even used as a low‐cost substitute for saffron in some regions. EFSA initially set its acceptable daily intake (ADI) at 0–7.5 mg/kg body weight, later increasing it to 0–10 mg/kg in 2016 following evaluations showing no evidence of adverse effects at relevant exposure levels (Zand et al. 2025). Estimated daily tartrazine intake in the general population varies considerably, ranging from 0.000671 to 14 mg per person depending on the country and the assessment method. Such differences largely reflect regional dietary habits; for example, overall consumption of synthetic dyes tends to be lower in Japan than in the United States (Ismail and Rashed 2022).

Tartrazine is characterized by highly polar sulfate groups. The molecule contains several potential reduction sites, with the azo (*N* = *N*) bond being the most susceptible to reductive cleavage. The stability of azo dyes depends on the presence of reducing agents that can break this bond and generate amines such as aniline, sulfanilic acid, and naphthoic acid, which may further degrade to ammonia. Metabolites of tartrazine can also generate reactive oxygen species (ROS) and contribute to oxidative stress (Erdemli et al. 2017). Reported reactions to tartrazine include allergic skin manifestations such as urticaria, angioedema, and eczema, as well as effects on the respiratory and gastrointestinal systems. Experimental studies, in turn, have indicated potential hepatotoxic, neurotoxic, reproductive toxic, and genotoxic effects under specific exposure conditions (Elhkim et al. 2007; Banc et al. 2024; Kavitha and Sangeetha 2025). However, several investigations have not detected some of these toxic effects, indicating variability in the reported outcomes (Amchova et al. 2024). Among studies that do report toxic effects, these responses have frequently been linked to tartrazine‐derived metabolites—most notably sulfanilic acid—which has been suggested as a contributing factor to the observed toxicity (Moutinho et al. 2007; Ismail and Rashed 2022; Barciela et al. 2023). However, studies comparing the toxicity of tartrazine and sulfanilic acid, as well as clarifying the contribution of this primary metabolite to overall toxicity, remain scarce.

As previously described, tartrazine has been associated with allergic skin reactions and respiratory involvement, suggesting that bronchial epithelial cells may represent a relevant target for evaluating its potential effects. For this reason, the BEAS‐2B cell line—an immortalized, non‐tumorigenic human bronchial epithelial model widely used in vitro to assess chemicals and biological agents with potential pulmonary toxicity or carcinogenic properties (Han et al. 2020)—was employed in the present study. In BEAS‐2B cells, the effects of tartrazine and its primary metabolite, sulfanilic acid, were examined by assessing cell viability with the MTT and neutral red uptake (NRU) assays, evaluating DNA damage through the alkaline comet assay, and analyzing apoptosis via annexin V/propidium iodide (PI) binding, caspase‐3/7 activity, and mitochondrial membrane potential (MMP).

## 2 Materials and Methods

### 2.1 Cell Culture and Preparation of Test Compounds

BEAS‐2B cells (Cytion, catalog no. 300311) were cultured in RPMI medium supplemented with 10% fetal bovine serum and 1% penicillin–streptomycin. Cells were maintained at 37°C in a humidified atmosphere containing 5% CO_2_. The culture medium was replaced twice per week, and cells were passaged once or twice weekly. All experiments were conducted using cells between passages 3 and 10.

Tartrazine (CAS Number: 1934‐21‐0, ≥ 85%) and sulfanilic acid (CAS Number: 121‐57‐3, Pharmaceutical Secondary Standard) were obtained from Sigma‐Aldrich (United States). Fresh stock solutions of both compounds were prepared in culture medium and sterilized using 0.22 μm membrane filters. Serial working dilutions were generated in the same medium. Cells maintained in culture medium alone served as the negative control, whereas positive controls were included in specific assays as described in the corresponding experimental procedures.

### 2.2 Assessment of Cell Viability Using MTT and NRU Assays

Cell viability was assessed using the MTT and neutral red uptake (NRU) assays. Before plating, cells were counted with a TC20 Automated Cell Counter (Bio‐Rad, USA), and their viability was verified by trypan blue exclusion. Cells were seeded into 96‐well plates at a density of 1 × 10^4^ cells per 100 μL and allowed to adhere for 24 h. Tartrazine and sulfanilic acid were then added at concentrations ranging from 1 to 5000 μM, followed by incubation for 24, 48, or 72 h. During tartrazine exposure experiments, before MTT and NRU incubation, the exposure media were removed, and cells were washed multiple times with PBS. Subsequently, freshly prepared dye solutions were added in compound‐free medium to avoid potential optical interference.

For the MTT assay, 100 μL of MTT working solution (0.5 mg/mL) was added to each well and incubated for 3 h. Formazan crystals formed in viable cells were subsequently solubilized with 100 μL DMSO, and absorbance was recorded at 540 nm. For the NRU assay, the culture medium was replaced with 200 μL of neutral red supplemented medium (50 μg/mL), and cells were incubated for 3 h. Following incubation, wells were washed and fixed with a formaldehyde–calcium solution, after which the incorporated dye was extracted using an acetic acid–ethanol mixture. Absorbance was then measured at 540 nm. All measurements were performed in three independent experiments, each carried out with four technical replicates per condition (Baysal and Atlı‐Eklioğlu 2021).

### 2.3 Apoptosis Assessment by Annexin V/PI, MMP, and Caspase‐3/7 Assays

To investigate early cell death responses, apoptosis was analyzed at 24 h, prior to the emergence of pronounced cytotoxic effects. Cells were exposed to selected sublethal concentrations of tartrazine (1–1000 μM), an elevated concentration (5000 μM), and sublethal concentrations of sulfanilic acid across the tested range (1–5000 μM).

BEAS‐2B cells were seeded at 3 × 10^5^ cells/6‐well plate, incubated for 24 h, and exposed to tartrazine or sulfanilic acid (1, 100, 1000, and 5000 μM) for 24 h. For Annexin V/PI analysis, cells were collected, adjusted to 1 × 10^6^ cells/mL, and stained with FITC Annexin V (2.5 μL) and PI (2.5 μL) using the Annexin V‐FITC/PI Apoptosis Detection Kit (Elabscience, China). After adding a 400‐μL binding buffer, samples were analyzed on a BD Accuri C6 Plus (FITC‐A, PerCP‐A). Flow cytometric analysis was performed by initially gating on cells based on forward and side scatter properties to exclude debris. Quadrant analysis of Annexin V–FITC and PI staining was then applied to distinguish viable (Annexin V^−^/PI^−^), early apoptotic (Annexin V^+^/PI^−^), late apoptotic (Annexin V^+^/PI^+^), and necrotic (Annexin V^−^/PI^+^) populations. Apoptosis was quantified as the sum of Annexin V^+^/PI^−^ and Annexin V^+^/PI^+^ populations.

MMP was evaluated using the JC‐1 Mitochondrial Membrane Potential Detection Kit (ABP Biosciences, United States). Cells (≤ 1 × 10^6^/mL, counted using trypan blue exclusion with a TC20 Automated Cell Counter (Bio‐Rad, United States)) were stained with JC‐1 for 20 min at 37°C; carbonyl cyanide 3‐chlorophenylhydrazone (CCCP; 25 mM, 1 μL, 10 min) served as a positive control. After washing, fluorescence was recorded on the BD Accuri C6 Plus (FITC‐A, PE‐A), and MMP was expressed as the red/green ratio. A flow cytometric analysis was performed by gating the main cell population based on FSC/SSC properties to exclude debris. Cells within the gate were analyzed for JC‐1 red and green fluorescence signals, and MMP was expressed as the red/green fluorescence ratio.

Caspase‐3/7 activity was assessed with the Cell Meter Caspase‐3/7 Activity Apoptosis Assay Kit (AAT Bioquest, United States). Cells (1 × 10^6^/mL, counted using trypan blue exclusion with a TC20 Automated Cell Counter (Bio‐Rad, United States)) were incubated with 1 μL Component A for 1 h at 37°C, washed, resuspended in an assay buffer, and stained with 1 μL PI for 10 min. Samples were analyzed on the BD Accuri C6 Plus (FITC‐A), and caspase‐3/7 activity was quantified based on the proportion of cells gated in the designated analysis region. Flow cytometric analysis was performed by gating the main cell population based on FSC/SSC properties to exclude debris. Caspase‐3/7 activity was quantified as the percentage of FITC‐positive cells within the defined analysis region.

### 2.4 Evaluation of DNA Damage by the Alkaline Comet Assay

DNA damage was assessed at 24 h to capture early genotoxic responses before the development of overt cytotoxicity. BEAS‐2B cells were exposed to sublethal concentrations of tartrazine and sulfanilic acid (1, 100, and 1000 μM), which maintained ≥ 75% cell viability after 24 h, and the alkaline comet assay was performed according to Tice et al. (2000) with minor modifications. BEAS‐2B cells were seeded at 5 × 10^4^ cells/well, exposed to tartrazine or sulfanilic acid for 24 h. A 75‐μM H_2_O_2_ treatment served as the positive control. Cell suspensions were embedded in low‐melting agarose (1%), lysed (for 45 min at 4°C), subjected to alkaline unwinding (for 15 min), and electrophoresed (25 V, 300 mA, 20 min). Slides were neutralized, stained with SYBR Green I, and analyzed using Comet Assay IV. For each treatment, 100 randomly selected nucleoids from two independent slides were scored, with hedgehog structures excluded. All analyses were performed in three independent experiments, and DNA damage was expressed as tail intensity (%) (Baysal and Atlı‐Eklioğlu 2021).

### 2.5 Statistical Analysis

Statistical analyses were performed in GraphPad Prism 9.0. Normality was tested with the Shapiro–Wilk test and variance homogeneity with the Brown–Forsythe test. Normally distributed and homogeneous data were analyzed by one‐way ANOVA with Dunnett's post hoc test; datasets with unequal variances were evaluated by Welch's ANOVA with Dunnett T3. Non‐normal data were analyzed using the Kruskal–Wallis test with Dunn's post hoc comparisons. Results are reported as mean ± SEM, and *p* < 0.05 was considered statistically significant.

## 3 Results

### 3.1 MTT and NRU Cell Viability Assays

**FIGURE 1:**
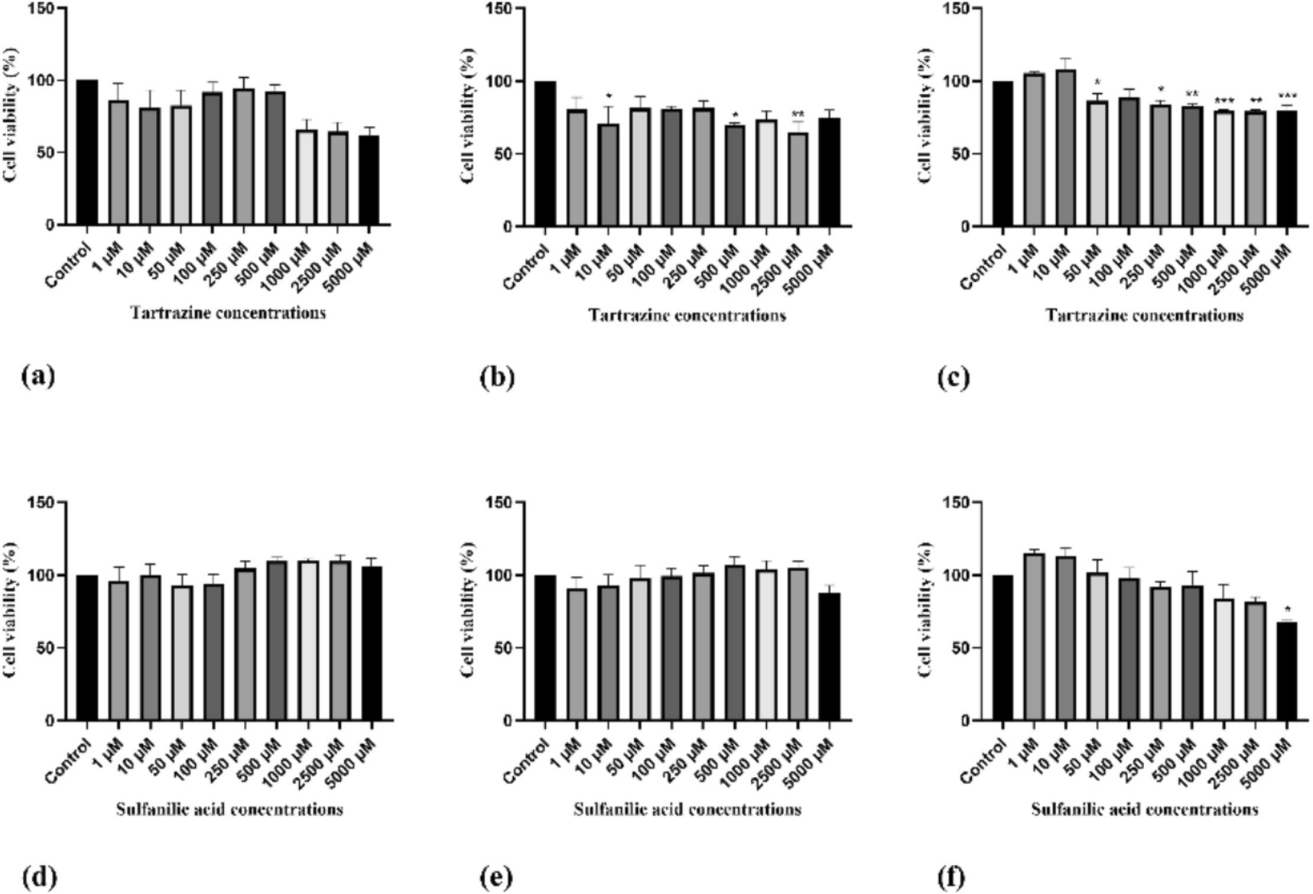
MTT‐based cell viability of BEAS‐2B cells exposed to tartrazine (a–c) and sulfanilic acid (d–f) at increasing concentrations. a and d correspond to 24 h, b and e to 48 h, and c and f to 72 h. Data represent mean ± SEM from three independent experiments. Statistical differences relative to the control are indicated. *p*‐value notation: GP 0.1234 (ns); 0.0332 (*); 0.0021 (**); 0.0002 (***); < 0.0001 (****).

The effects of tartrazine and sulfanilic acid on the viability of BEAS‐2B cells were evaluated using the MTT and NR assays across a concentration range of 1–5000 μM following 24, 48, and 72 h of exposure. According to the MTT results (Figure 1), tartrazine induced a concentration‐ and time‐dependent reduction in cell viability, with the decreases observed at 48 and 72 h being statistically significant compared with the control group. In contrast, sulfanilic acid did not cause notable changes in cell viability under the same experimental conditions, except for a significant decrease observed at the highest concentration after 72 h of exposure.

**FIGURE 2:**
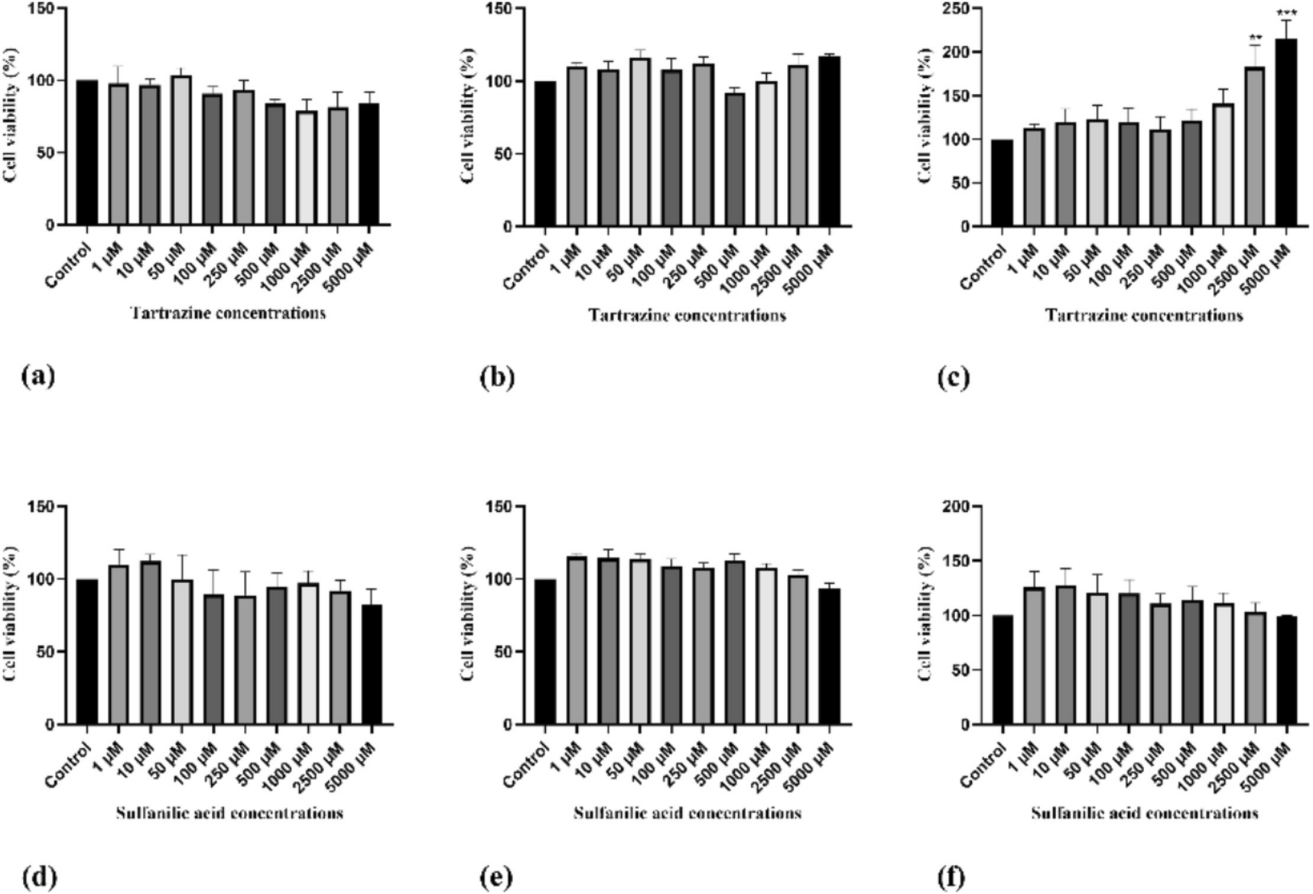
NRU assay–based evaluation of BEAS‐2B cell viability following exposure to tartrazine (a–c) and sulfanilic acid (d–f) at multiple concentrations. a and d represent 24 h, b and e correspond to 48 h, and c and f illustrate 72 h exposure. Values are shown as mean ± SEM from three independent experiments. Statistical comparisons with the control group are indicated. *p*‐value notation (GraphPad): 0.1234 (ns); 0.0332 (*); 0.0021 (**); 0.0002 (***); < 0.0001 (****).

According to the NRU assay findings (Figure 2), neither tartrazine nor sulfanilic acid produced significant alterations in cell viability. Notably, however, tartrazine induced statistically significant increases in viability at the two highest concentrations following 72 h of exposure when compared with the control group.

### 3.2 Apoptosis Analysis by Annexin V/PI, MMP, and Caspase‐3/7 Assays

**FIGURE 3:**
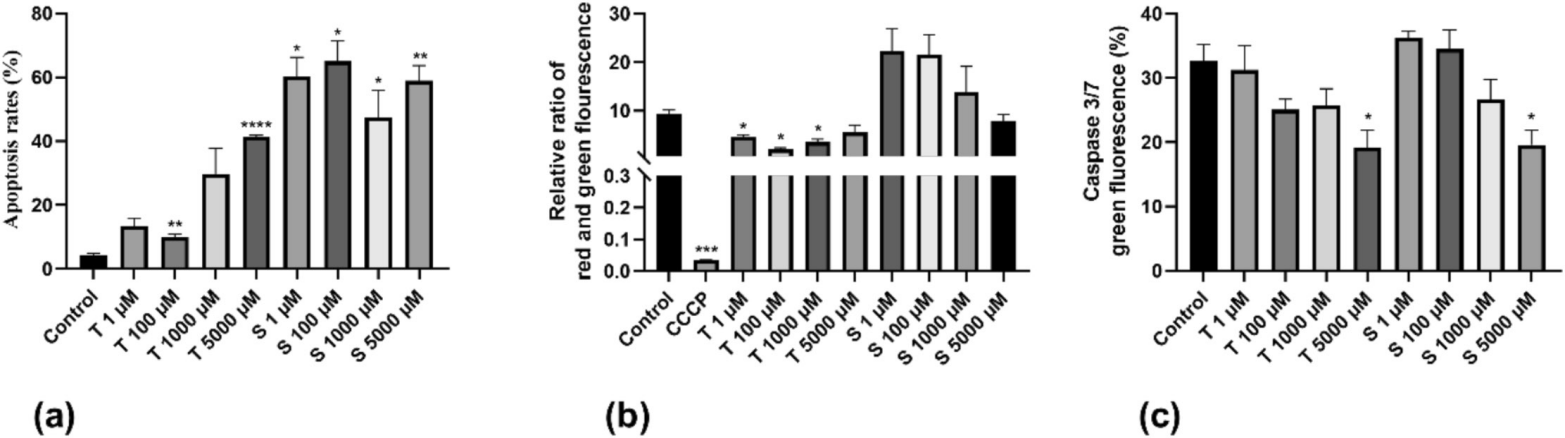
Apoptotic responses of BEAS‐2B cells following 24 h exposure to selected concentrations of tartrazine (T) and sulfanilic acid (S). Apoptosis was assessed using annexin V/PI staining (a), MMP analysis with JC‐1 dye (b), and caspase‐3/7 activity measurement (c). Data represent mean ± SEM from three independent experiments for Annexin V/PI analysis and two independent experiments for MMP and caspase‐3/7 assays. Statistical differences relative to the control group are indicated. *p*‐value notation: GP 0.1234 (ns); 0.0332 (*); 0.0021 (**); 0.0002 (***); < 0.0001 (****).

Predominantly sublethal concentrations of tartrazine (1, 100, and 1000 μM), together with a higher concentration (5000 μM), and sublethal concentrations of sulfanilic acid (1–5000 μM) were tested in BEAS‐2B cells. Following 24 h of exposure, increases in apoptotic cell ratios were observed, and these increases were statistically significant at 100 and 5000 μM for tartrazine, whereas sulfanilic acid induced statistically significant increases at all tested concentrations. These findings demonstrate that both compounds triggered apoptosis under the present experimental conditions (Figure 3a).

In experiments evaluating MMP, the results confirmed that the positive control CCCP caused a pronounced decrease in MMP levels. Tartrazine exposure also led to reductions in MMP, with the decreases at 1, 100, and 1000 μM being statistically significant compared with the control. In contrast, sulfanilic acid exposure did not result in significant changes in MMP levels (Figure 3b).

Contrary to expectations, caspase‐3/7 levels decreased with increasing concentration, and this reduction was statistically significant at the highest concentrations of both compounds (Figure 3c).

### 3.3 DNA Damage Analysis by Alkaline Comet Assay

**FIGURE 4:**
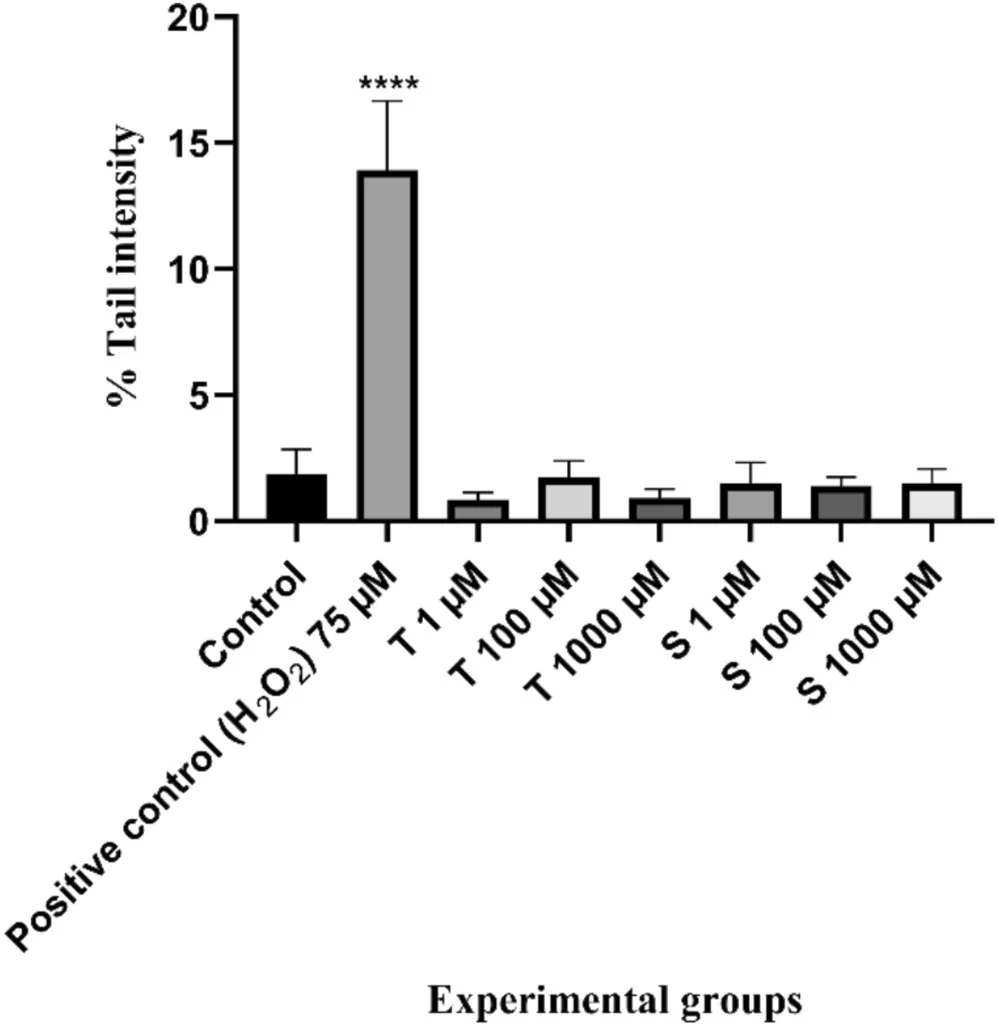
DNA damage in BEAS‐2B cells after 24 h exposure to selected concentrations of tartrazine (T) and sulfanilic acid (S), assessed using the alkaline comet assay. Data are expressed as mean ± SEM from three independent experiments. Statistical differences relative to the control group are indicated. *p*‐value notation (GraphPad): 0.1234 (ns); 0.0332 (*); 0.0021 (**); 0.0002 (***); < 0.0001 (****).

DNA damage was evaluated in BEAS‐2B cells exposed to 1, 100, and 1000 μM concentrations of tartrazine and sulfanilic acid for 24 h using the comet assay. The positive control H_2_O_2_ (75 μM) induced a significant increase in tail intensity percentage compared with the control group. At the tested sublethal concentrations, neither tartrazine nor sulfanilic acid induced significant changes in tail intensity (%) compared with the control group (Figure 4).

## 4 Discussion

Azo dyes, comprising nearly half of the organic colorants used across food, pharmaceutical, and cosmetic industries, are synthetic compounds with purely aesthetic functions and no nutritional value. Tartrazine is one of the most widely used azo dyes owing to its vivid coloration and low cost (Madesh et al. 2026). It is a widely used synthetic azo dye present in numerous processed foods and beverages—permitted up to 100 mg/kg in the European Union—and is also incorporated into pharmaceuticals, cosmetics, and textiles, leading to chronic human exposure through diet, dermal contact, and inhalation (Hoang and Park 2026).

Tartrazine has been reported to induce a wide spectrum of toxicological effects, including allergic and hypersensitivity reactions, immunosuppression, biochemical and organ dysfunction (notably hepatic, renal, and reproductive), cytotoxicity, genotoxicity, neurobehavioral alterations, and even growth impairment and lethality under certain dietary conditions (Sultana et al. 2023). Tartrazine's azo bond undergoes microbial reductive cleavage to form aromatic amines such as sulfanilic acid, whose metabolites can generate ROS and induce oxidative stress, thereby disrupting organ structure and metabolism in tissues including the liver, kidney, and stomach (Hussein et al. 2025). However, the presence of studies reporting minimal or no toxicity associated with tartrazine keeps the issue open to debate (Visternicu et al. 2025). Moreover, toxicity data for sulfanilic acid—one of the primary metabolites presumed to contribute to tartrazine's toxic effects—remain considerably limited. Sulfanilic acid may act as a secondary exposure agent following systemic distribution, thereby enabling interaction with tissues that are not primarily adapted to azo dye exposure. In this context, the BEAS‐2B bronchial epithelial cell model may represent a relevant non‐tumorigenic system for investigating cellular responses to both tartrazine and its metabolite under alternative exposure scenarios, including inhalation and occupational contact. Therefore, in this study, BEAS‐2B cells were employed to assess the effects of tartrazine and its one of main metabolite sulfanilic acid using MTT and NRU assays for cell viability, the alkaline comet assay for DNA damage, annexin V/PI staining, caspase‐3/7 activity, and MMP for apoptosis.

The concentration range used in this study was selected to encompass a broad experimental spectrum in order to characterize concentration‐dependent cytotoxic and mechanistic cellular responses under controlled in vitro conditions. Comparable high concentration ranges have been reported in previous in vitro toxicological studies investigating tartrazine, with maximum concentrations reaching 8 mM (Mpountoukas et al. 2010), 2500 μg/mL (approximately 4680 μM; Atlı Şekeroğlu et al. 2017), and 2000 μg/mL (approximately 3740 μM; Pasdaran et al. 2022). These studies similarly aimed to define cellular response thresholds rather than directly replicate physiological exposure scenarios. In contrast, estimated daily intake values (0.000671–14 mg/person/day; Ismail and Rashed 2022) and biomonitoring data showing urinary tartrazine levels in the low nanogram per milliliter range (approximately 0.04–109.40 ng/mL; Lei et al. 2013) indicate that typical human exposure corresponds to submicromolar or lower internal concentrations. Thus, although the highest concentrations tested exceed estimated human exposure levels by several orders of magnitude, they were intentionally included to enable hazard identification and mechanistic exploration by revealing concentration‐dependent cellular response patterns across a wide range. In addition, the tartrazine used in this study complied with EFSA specifications for food‐grade material (EFSA ANS Panel 2009). As such, minor subsidiary coloring components and inorganic salts may be present within regulated limits and cannot be entirely excluded as contributors to the observed effects. However, this composition more closely reflects realistic exposure conditions compared with analytical‐grade preparations and may therefore better represent the complexity of real‐world tartrazine exposure.

Because each assay has inherent methodological differences and may respond variably to specific test compounds, evaluating cell viability with more than one complementary assay is generally considered beneficial. In this study, cell viability was assessed using the MTT assay, which quantifies metabolic competence through mitochondrial succinate and NAD(P)H‐dependent dehydrogenase–mediated reduction of MTT to insoluble formazan crystals, and the NRU assay, which evaluates membrane integrity and lysosomal functionality based on the active accumulation and retention of the cationic vital dye neutral red (Kamiloglu et al. 2020). Tartrazine reduced BEAS‐2B viability in a concentration‐ and time‐dependent manner in the MTT assay, whereas sulfanilic acid showed minimal effects except at the highest dose after 72 h; however, neither compound caused significant changes in the NRU assay, aside from modest viability increases at the highest two tartrazine concentrations at 72 h. MTT results suggest a clearer cytotoxic profile for tartrazine than for its metabolite, while the overall lack of NRU alterations indicates that tartrazine and sulfanilic acid did not exert notable lysosome‐directed toxicity under the tested conditions. However, the viability elevations observed at high tartrazine concentrations in the NRU assay at 72 h may indicate tartrazine‐induced alterations in lysosomal function, as chemicals acting locally on lysosomes are known to produce either elevated or diminished NRU (Repetto et al. 2008). In addition, a previous study reported that tartrazine may induce oxidative stress and histopathological alterations, including cytoplasmic vacuolation (Amin et al. 2010). Such vacuolar changes, which can be associated with autophagic or degenerative processes, may increase intracellular acidic compartments and thereby enhance Neutral Red accumulation. Accordingly, the observed NRU assay (increased optical density at 72 h) may suggest alterations in lysosomal–vacuolar dynamics rather than true increases in cell viability. Previous in vitro studies generally describe tartrazine as exhibiting limited cytotoxicity; however, reported outcomes vary considerably depending on the cellular model and experimental conditions employed. While no reduction in cell viability has been observed in certain human‐derived cell types, including fibroblasts (Pasdaran et al. 2022) and leukocytes (Floriano et al. 2018), other studies have reported cytotoxic effects under specific conditions. Notably, exposure of Chinese hamster ovary (CHO) cells to tartrazine resulted in marked alterations in oxidative stress–related parameters, accompanied by evidence of cytotoxicity (Demirkol et al. 2012), and cytotoxic effects have been observed at high concentrations and in the absence of metabolic activation in human lymphocyte cultures (Atlı Şekeroğlu et al. 2017). In contrast, data on sulfanilic acid remain comparatively scarce. Available evidence suggests its potential to induce oxidative stress–related alterations and functional disturbances in selected experimental models (Ameur et al. 2018; Himri et al. 2011), whereas the absence of cytotoxicity reported in some human‐derived cell lines supports the notion of cell‐type–dependent biological effects (Himri et al. 2013).

Cell death plays roles in both normal physiological processes and pathological conditions (Bertheloot et al. 2021). Apoptosis enables the regulated removal of damaged cells and supports tissue homeostasis (Mustafa et al. 2024) and may represent one of the key mechanisms involved in chemical‐induced toxicity (Gomez‐Lechon 2002). Annexin V binding is widely applied as a sensitive approach for the detection of apoptotic cells at different stages and for their distinction from necrotic cells; in addition, because caspase‐3 and caspase‐7 play pivotal roles in apoptotic phenotypic alterations, assessment of caspase activity is commonly used to differentiate apoptosis from the majority of necrotic cell death pathways, with the exception of pyroptosis (Costigan et al. 2023). Furthermore, changes in MMP during apoptosis provide additional insight into cell death mechanisms and are commonly assessed using JC‐1 (Troiano et al. 2007). In this study, exposure of BEAS‐2B cells to selected concentrations of tartrazine and sulfanilic acid resulted in increased apoptotic cell ratios after 24 h. Tartrazine induced statistically significant increases in apoptosis at some of the tested concentrations, whereas sulfanilic acid produced significant increases across all tested concentrations. Assessment of MMP showed that tartrazine caused a significant reduction in MMP, while sulfanilic acid did not induce significant changes. In contrast, caspase‐3/7 activity decreased at higher concentrations of both compounds despite the observed increases in apoptotic cell populations. The findings of this study indicate that, under the tested conditions, cell death is not restricted to a single mechanism but may involve the concurrent or sequential activation of multiple cell death pathways in a concentration‐dependent manner. Although caspase activation is often associated with apoptosis, growing evidence suggests that caspases are not invariably required for the execution of cell death and that reduced caspase activity may reflect a shift in the mode of cell death rather than its suppression (Kögel and Prehn 2013; Kroemer and Martin 2005). In this context, the decrease in caspase‐3/7 activity observed at higher concentrations, together with alterations in MMP, suggests the involvement of caspase‐independent mechanisms without implying complete loss of mitochondrial function or cellular energy status. Mitochondrial membrane disruption may facilitate the release of pro‐apoptotic factors such as apoptosis‐inducing factor (AIF), which has been reported to promote chromatin condensation and DNA fragmentation independently of caspase activation. Accordingly, the apoptotic phenotype observed here may reflect engagement of mitochondrial, caspase‐independent pathways rather than classical executioner caspase signaling (Candé et al. 2002). Moreover, oxidative stress has been reported to modulate caspase activity through oxidation or S‐glutathiolation of catalytic cysteine residues, which may lead to reduced caspase activity despite ongoing apoptotic signaling (Redza‐Dutordoir and Averill‐Bates 2016). Although ROS generation was not directly assessed in the present study, tartrazine has been reported to induce oxidative stress in previous studies (Visternicu et al. 2025); therefore, ROS‐mediated modulation of mitochondrial function and caspase activity may represent a plausible contributing mechanism to the observed cellular responses. On the other hand, the Annexin V–positive/MMP‐preserved profile observed for sulfanilic acid may reflect an early apoptotic stage occurring upstream of mitochondrial depolarization. Previous kinetic studies have demonstrated that phosphatidylserine externalization can precede loss of MMP, indicating initiation of apoptotic signaling at the plasma membrane level before mitochondrial involvement (Denecker et al. 2000). Moreover, because metabolic viability was preserved (MTT/NRU) and no necrotic shift was observed (data not shown), these findings may be indicative of a reversible stress response. In line with the concept of anastasis, early apoptotic features may not necessarily commit cells to irreversible death but could instead represent a transient, recoverable state under sublethal conditions (Zakharov et al. 2020). Consistent with the context‐dependent effects of tartrazine, in vivo studies have demonstrated that chronic exposure can induce oxidative stress–associated apoptotic signaling involving caspase‐3 in neural tissues (Essawy et al. 2023). Additionally, tartrazine exposure has been shown to disrupt mitochondrial homeostasis and modulate the expression of key apoptotic regulators, including Bcl‐2 family members and p53, in a developmental neurotoxicity model using zebrafish embryos (Haridevamuthu et al. 2024). Despite the scarcity of data on sulfanilic acid, evidence from one study reporting mitochondrial depolarization at 1 mM in pancreatic AR42J cells suggests a possible role for this azo dye metabolite in perturbing mitochondrial homeostasis (Ameur et al. 2018).

Preservation of genomic sequence integrity is essential for the continuity of life in living organisms. DNA is constantly challenged by damaging agents that can compromise genomic stability and contribute to the development of disease. Given that routinely used biological, physical, and chemical agents can adversely affect human health, systematic evaluation of their genotoxic potential and appropriate regulation of their use are of critical importance (Chatterjee and Walker 2017). The comet assay is a widely applied and sensitive method in genotoxicity testing, enabling the detection of DNA strand breaks and alkali‐labile sites at the single‐cell level in virtually all types of eukaryotic cells (Collins et al. 2023). In this study, comet assay analysis showed that, unlike the positive control H_2_O_2_, exposure of BEAS‐2B cells to sublethal concentrations of tartrazine or sulfanilic acid in DNA strand breaks, indicating no detectable DNA damage under the experimental conditions tested. Reports on the genotoxicity of tartrazine are conflicting, with both positive and negative findings depending on experimental design, exposure conditions, and biological model. In contrast, information on the genotoxic potential of its primary metabolite, sulfanilic acid, is limited. Within this context, a study employing chromosome aberration analysis and the cytokinesis‐block micronucleus cytome assay reported no evidence of genotoxicity in human lymphocytes exposed to tartrazine at the tested concentrations (Haverić et al. 2017). Similarly, an in vivo genotoxicity study conducted in accordance with OECD guidelines demonstrated no genotoxic activity of tartrazine in the bone marrow micronucleus assay or in comet assays performed in the liver, stomach, and colon (Bastaki et al. 2017). In contrast, an in vitro study showed that tartrazine induced DNA damage in human lymphocytes at concentrations ranging from 0.25 to 64.0 mM, as assessed by the alkaline comet assay, despite the absence of cytotoxic effects (Soares et al. 2015). Moreover, comet assay analysis in multiple tissues of ddY mice identified tartrazine among food dyes that induced dose‐related DNA damage in gastrointestinal organs, including the colon, at doses approaching acceptable daily intake levels (Sasaki et al. 2002).

Overall, the present data suggest that tartrazine and its primary metabolite, sulfanilic acid, can affect cell viability and apoptosis‐related responses in BEAS‐2B bronchial epithelial cells under the experimental conditions applied. The increase in apoptotic cell populations, together with observed changes in MMP and caspase‐3/7 activity, points to the involvement of cell death–associated processes; however, these outcomes were evaluated using a limited number of apoptotic endpoints. Likewise, the lack of detectable DNA damage as determined by the alkaline comet assay reflects the scope of a single genotoxicity endpoint and does not preclude the existence of other forms of genomic or chromosomal alterations. Accordingly, while these findings provide useful insight into cellular responses to tartrazine and sulfanilic acid at sublethal concentrations, additional investigations employing a broader range of genotoxicity assays and cell death markers would be necessary to more fully define their toxicological profiles.

## 5 Conclusion

In conclusion, this study shows that tartrazine reduces cell viability and induces apoptotic responses in BEAS‐2B bronchial epithelial cells, while its metabolite sulfanilic acid exerts comparatively milder cytotoxic effects. At sublethal concentrations, neither compound caused detectable DNA damage as assessed by the alkaline comet assay. These results suggest that the cellular effects of tartrazine and sulfanilic acid in BEAS‐2B cells are primarily associated with alterations in cell viability and apoptosis‐related processes, while no significant DNA strand breaks were detected under the experimental conditions tested.

## Conflicts of Interest

The authors declare no conflicts of interest.

## Data Availability

The data that support the findings of this study are available from the corresponding author upon reasonable request.
